# Compare the accuracy and precision of Coulter LH780, Mindray BC-6000 Plus, and Sysmex XN-9000 with the international reference flow cytometric method in platelet counting

**DOI:** 10.1371/journal.pone.0217298

**Published:** 2019-05-24

**Authors:** Yi Sun, Zuojian Hu, Zhili Huang, Huaping Chen, Shanzi Qin, Zhong Jianing, Siyuan Chen, Xue Qin, Yi Ye, Chengbin Wang

**Affiliations:** 1 Department of Clinical Laboratory, Chinese People’s Liberation Army General Hospital, Beijing, China; 2 Department of Laboratory Medicine, First Affiliated Hospital of Guangxi Medical University, Nanning, Guangxi, China; 3 Department of Blood Transfusion, Second Affiliated Hospital of Guangxi Medical University, Nanning, Guangxi, China; 4 Hematology Product Development Department, Shenzhen Mindray Bio-Medical Electronics Co., Ltd, Shenzhen, China; Inselspital Universitatsspital Bern, SWITZERLAND

## Abstract

**Objective:**

The aim of this study is to evaluate the performance of different platelet counting methods (optical, impedance, fluorescence and hand counting) applied in different analysers by comparing with the international flow cytometric reference method (IRM).

**Methods:**

A total of 333 blood samples from different subgroups (168 cases with thrombocytopenia, 136 cases with normal platelet counts and 29 cases with thrombocytosis) were tested. Regarding IRM as the gold standard, we compared the accuracy and precision of different platelet count methods; i.e. LH780 (impedance), BC-6000 Plus (optical (O) and impedance (I)), Sysmex XN-9000 (optical (O), impedance (I), fluorescence (F)), and hand counting.

**Results:**

Sysmex XN-9000-F (r = 0.988) had the best correlation with IRM for thrombocytopenic samples; BC-6000 Plus-I (r = 0.966) was more relevant to IRM than any other method for samples with normal platelet counts. Correlation between Sysmex XN-9000-I (r = 0.960) and IRM was the highest among these methods for samples with thrombocytosis. For bias evaluation, the average bias of Sysmex XN-9000-F was -1.5 × 10^9^/L (95% LA = -9.4 to +6.4) for samples with thrombocytopenia, compared with IRM. BC-6000 Plus-I had a small mean difference with IRM for samples with normal platelet counts or thrombocytosis. Moreover, all evaluated methods had acceptable sensitivity, specificity, and concordance rates as compared with IRM in the diagnosis of thrombocytopenia and thrombocytosis.

**Conclusions:**

Platelet counting by Sysmex XN-9000-F is more accurate than other methods for thrombocytopenic samples. BC-6000 Plus-I has superior association and consistency for normal platelet counts. As for thrombocytosis patients, Sysmex XN-9000-I has the highest correlation with IRM while Sysmex XN-9000-O has the highest diagnosis efficacy.

## 1. Introduction

The accuracy of platelet count is critical for clinical laboratories and clinicians to assess the risk of bleeding in patients [1]. Over the years, platelet counting has been performed by microscopic counting, automated blood cell analyzers, and the international reference method based on flow cytometry proposed by International Council for Standardization in Haematology (ICSH) [2]. The impedance method is a classic platelet counting method invented by Beckman Coulter [3]; but it has some limitations, such as being unable to distinguish platelets from particles whose size and volume are similar to platelets. Thus, many instrument manufacturers have applied a variety of measures to solve this problem. Advia 120, Cell-Dyn 4000, Mindray BC-6800 and Abbot Diagnostics are platelet counters using the optical counting method that can differentiate blood components according to the type and strength of light signals [4–6]. Another analyzer, the Sysmex XN-3000, is designed to use the method of fluorescent nucleic acid staining to count platelets [7]. The above methods have improved the ability of automatic blood analyzers to identify platelets.

Decreased platelet count is seen in thrombocytopenic purpura, aplastic anemia, leukemia, etc [8]. The increased number of platelets can be seen in primary thrombocythemia, polycythemia vera and chronic myelogenous leukemia [9, 10]. Therefore, accurate counting of platelets contributes to the diagnosis and differential diagnosis of clinical hemostasis and thrombotic diseases. A comparative study about the accuracy and reliability of different platelet count methods will help clinicians choose the most pratical one for patients in different conditions [11]. As far as we know, there are no comparative studies on various platelet counting methods (LH780, BC-6000 Plus-O, BC-6000 Plus-I, Sysmex XN-9000-O, Sysmex XN-9000-I, Sysmex XN-9000-F, and hand counting) at different platelet levels (thrombocytopenia, normal and thrombocytosis). The purpose of this study is to evaluate the accuracy and precision of different platelet counting methods (optical, impedance, fluorescence and hand counting) in different analysers by comparing with IRM. The detecting parameters, including sensitivity, specificity, and coincidence rate, were calculated for samples from patients with thrombocytopenia, thrombocytosis, or normal platelet level.

## 2. Materials and methods

### 2.1 Methods

A total of 410 whole blood samples were collected, of which 77 were excluded because they met any of the exclusion criteria. Finally, 333 dipotassium ethylenediaminetetraacetic acid (K_2_-EDTA)-anticoagulated blood samples with platelets ranging from 1.88 × 10^9^/L to 960 × 10^9^/L were selected for this study; the number of platelets was counted by IRM method. All blood samples were obtained from the pediatric ward, oncology ward, and hematology ward of the inpatient department of the First Affiliated Hospital of Guangxi Medical University. All specimens are leftover samples collected from October 2017 to February 2018. All specimens were tested by different detection methods parallelly within 6 hours after the blood samples were collected. Authors were blinded and had no access to information that could identify individual participants during or after data collection. The exclusion criteria in this study were as follows: (i) Automated blood analyzers performing "flags" information: possible presence of platelet clumps or other substances that may interfere with automatic analysis; (ii) Peripheral blood smears that showed platelet clumps, giant platelets, red blood cell fragments, or microcytosis. This study was approved by the Ethics Committee of the First Affiliated Hospital of Guangxi Medical University.

Eight platelet count methods were evaluated in parallel: Beckman Coulter LH780 Impedance (LH780), Mindray BC-6000 Plus-optical (BC-6000 Plus-O), Mindray BC-6000 Plus-impedance (BC-6000 Plus-I), Sysmex XN-9000- optical (Sysmex XN-9000-O), Sysmex XN-9000- impedance (Sysmex XN-9000-I), Sysmex XN-9000- fluorescence (Sysmex XN-9000-F), Hand counting, IRM.

Based on the International Council for Standardization in Haematology (ICSH) guidelines and the International Society of Laboratory Hematology, the IRM method (BD FACS Calibur flow cytometry, BD Pharmingen reagents, PE Mouse Anti-Human CD41/CD61) for platelet counting is briefly described as follows: 5 μl of mixed blood sample, 100 μl of phosphate buffered saline with bovine serum albumin (PBS-BSA), 5 μl of anti-CD41 antibody and 5 μl of anti-CD61 antibody were added into the flow cytometry-specific reaction tube; after thoroughly mixing, the sample was incubated at room temperature for 15 minutes in the dark. Finally, a diluted sample of 1:1000 was prepared by adding 4.85 mL of PBS-BSA. A minimum of 50,000 red blood cell (RBC) events or 1000 platelet events were counted by flow cytometry. The final calculation formula of platelets was as follows:

Plateletcount=(*RBCcount)/R

The *RBC count here represents the mean red blood cell count measured by LH780 (impedance). R = RBC gated events/platelet gated events.

According to the National Guide to Clinical Laboratory Procedures (Third Edition) [12], the operation steps of the hand counting method are briefly described as follows: 20 μl of blood specimens and 0.38 ml of aqueous ammonium oxalate solution were added into a clean glass test tube; after mixing well, a drop of platelet suspension was placed in a Neubauer hemocytometer and allowed to stand for 15 min. The final platelet count results are obtained from the formula below:

$$\text{Platelet}\;\text{count}=\text{N}\times 5\times 10\times 20\times 10^{6}/\text{L}$$

N represents the total platelets of five small squares in the middle and four corners of the large square in the middle of the Neubauer hemocytometer. ×5 represents the conversion of 5 squares into a large square. ×10 represents a large square volume of 0.1μl converted to 1μl. ×200 represents the actual dilution factor of blood. ×10^6^ represents conversion from 1μl to 1L.

### 2.2 Quality control

According to the manufacturer’s guidelines, the instrument was calibrated using the calibration samples supplied by the manufacturer. The high, normal and low control samples were run in line with the National Accreditation Board for Laboratories (NABL) guidelines.

### 2.3 Coefficient of variation

In order to assess the inaccuracy and the coefficient of variation of different methods, a single sample was run six times using each different method. The mean platelet count of the sample which was used to assess the coefficient of variation (CV) was 240.9 × 10^9^/L.

### 2.4 Statistical analysis

Data analysis and image production were achieved by using SPSS version 17.0 (SPSS Inc., Chicago, IL, USA), Analyse IT and MedCalc statistical software (version 11.3.8.0). The Spearman rank correlation coefficient was used to assess the correlation between different detection methods and IRM. The consistency between different measurement methods and IRM was evaluated by Bland-Altman analysis. The results of the Bland-Altman analysis were expressed as mean difference, which was the average difference between counts from each method and IRM, with 95% limits of agreement (95% LA). The LA was defined as -1.96s to +1.96s. Using IRM as the gold standard, the sensitivity, specificity and coincidence rate of different methods for counting platelets were analyzed by Inter-rater agreement (Kappa) test. The Kolmogorov-Smirnov test was used to evaluate the distribution status of the obtained data. The level of *P* < 0.05 (two-tailed) was considered to be statistically significant.

## 3. Results and discussion

### 3.1 Descriptive analysis of different methods for platelet counting

As shown in Table 1, the IRM method had a minimum CV value of 1.51%, while that of hand counting method reached 18.39%. In addition, CV values came from impedance counting method were lower than those from optical method in both Mindray BC-6000 Plus and Sysmex XN-9000 systems.

**Table 1:** Descriptive analysis of different methods for platelet counting.

Method	Minimum	Maximum	Mean	CV%
LH780	4.60	986.5	166.48	8.75%
BC-6000 Plus-O	4.00	1033.5	178.04	7.28%
BC-6000 Plus-I	2.00	987.5	172.92	3.26%
Sysmex XN-9000-O	6.00	945.0	159.23	9.76%
Sysmex XN-9000-I	4.00	965.0	175.03	6.33%
Sysmex XN-9000-F	4.00	959.0	160.19	6.74%
Hand counting	4.20	980.0	163.93	18.39%
IRM	1.88	960.0	169.64	1.51%

### 3.2 Comparison between different platelet measurements and IRM

Based on the clinical significance of platelet level, patients were divided into three groups: platelet counting by IRM was less than 100 × 10^9^/L (thrombocytopenia), platelet counting by IRM was between 100 × 10^9^/L and 450 × 10^9^/L (normal), and platelet counting by IRM was higher than 450 × 10^9^/L (thrombocytosis). The results of Spearman rank correlation and Bland-Altman analysis are presented in Table 2. For thrombocytopenic samples (S1 and S4 Figs), Sysmex XN-9000-F showed the superior correlation (r = 0.988) and agreement (average bias = -1.5) with IRM. BC- 6000 Plus-I had the best association (r = 0.966) and consistency (average bias = +4.5) with IRM for platelet counts in the normal range (S2 and S5 Figs). However, compared with other evaluated methods (LH780, BC-6000 Plus, and Sysmex XN-9000), the correlation between hand counting method and IRM was weaker for samples with thrombocytopenia or normal platelet counts. For samples with thrombocytosis (S3 and S6 Figs), the results of Sysmex XN-9000-I (r = 0.960) were highly correlated with those of IRM, while BC-6000 Plus-I (average bias = +0.4) had a minimal mean difference with IRM.

**Table 2:** Spearman rank correlation and Bland-Altman analysis of platelets between evaluated methods and IRM.

Levels of platelets	N	r	*P*	Average bias (mean difference)	95% Limits of agreement
**<100×10**^**9**^**/L**	**168**				
LH780 vs IRM		0.964	0.000	+3.8	-10.2 to +17.8
BC-6000 Plus-O vs IRM		0.985	0.000	+4.3	-5.5 to +14.1
BC-6000 Plus-I vs IRM		0.961	0.000	+2.8	-11.7 to +17.3
Sysmex XN-9000-O vs IRM		0.984	0.000	-1.5	-10.8 to +7.8
Sysmex XN-9000-I vs IRM		0.937	0.000	+4.9	-14.9 to +24.7
Sysmex XN-9000-F vs IRM		0.988	0.000	-1.5	-9.4 to +6.4
Hand counting vs IRM		0.922	0.000	+3.1	-25.8 to +32.1
**100×10**^**9**^**/L-450×10**^**9**^**/L**	**136**				
LH780 vs IRM		0.947	0.000	-7.6	-67.4 to +52.2
BC-6000 Plus-O vs IRM		0.961	0.000	+9.0	-42.6 to +60.6
BC-6000 Plus-I vs IRM		0.966	0.000	+4.5	-42.2 to +51.1
Sysmex XN-9000-O vs IRM		0.962	0.000	-16.6	-69.8 to +36.6
Sysmex XN-9000-I vs IRM		0.947	0.000	+8.7	-48.0 to +65.5
Sysmex XN-9000-F vs IRM		0.959	0.000	-15.5	-67.0 to +36.0
Hand counting vs IRM		0.841	0.000	-9.7	-101.3 to +81.9
**>450×10**^**9**^**/L**	**29**				
LH780 vs IRM		0.855	0.000	-22.7	-136.4 to +91.0
BC-6000 Plus-O vs IRM		0.876	0.000	+29.5	-77.2 to +136.1
BC-6000 Plus-I vs IRM		0.932	0.000	+0.4	-74.5 to +75.4
Sysmex XN-9000-O vs IRM		0.864	0.000	-33.4	-139.6 to +72.9
Sysmex XN-9000-I vs IRM		0.960	0.000	-7.4	-66.6 to +51.7
Sysmex XN-9000-F vs IRM		0.831	0.000	-26.9	-153.4 to +99.5
Hand counting vs IRM		0.851	0.000	-38.0	-173.0 to +97.0

### 3.3 Evaluation of the test characteristics of different platelet detection methods

Regarding IRM as the gold standard, our study obtained the true positive (TP), false positive (FP), true negative (TN), false negative (FN), and Kappa value of evaluated methods at designated cutoff values by using Kappa analysis, as shown in Table 3. Sensitivity, specificity, and coincidence rate were obtained by the following calculation: sensitivity = TP/ (TP + FN), specificity = TN/ (TN + FP), and coincidence rate = (TP + TN)/ (TP + FP + TN + FN). When compared with IRM, all evaluated methods had acceptable sensitivity, specificity, and concordance rates in the diagnosis of thrombocytopenia and thrombocytosis. Sysmex XN-9000-F had the highest degree of agreement for thrombocytopenic samples, while Sysmex XN-9000-O showed the best sensitivity, specificity, concordance rate, and Kappa value for samples with thrombocytosis.

**Table 3:** Taking IRM as the gold standard, the detection characteristics of the evaluated methods.

Cutoff	Sensitivity	Specificity	Concordance Rate	Kappa value (95% CI of K)
**<100×10**^**9**^**/L**				
LH780	97.6% (0.94–0.99)	98.8% (0.96–1.00)	98.2% (0.96–0.99)	0.964(0.935–0.993)
BC-6000 Plus-O	94.0% (0.89–0.97)	100% (0.98–1.00)	97.0% (0.94–0.98)	0.940(0.903–0.977)
BC-6000 Plus-I	96.4% (0.92–0.99)	98.8% (0.96–1.00)	97.6% (0.95–0.99)	0.952(0.919–0.985)
Sysmex XN-9000-O	99.4% (0.97–1.00)	98.2% (0.94–1.00)	98.8% (0.96–1.00)	0.976(0.953–0.999)
Sysmex XN-9000-I	96.4% (0.92–0.99)	98.2% (0.94–1.00)	97.3% (0.95–0.99)	0.946(0.911–0.981)
Sysmex XN-9000-F	100% (0.98–1.00)	99.4% (0.97–1.00)	99.7% (0.98–1.00)	0.994(0.982–1.000)
Hand counting	94.6% (0.90–0.98)	93.9% (0.89–0.97)	94.3% (0.91–0.96)	0.886(0.836–0.936)
**>450×10**^**9**^**/L**				
LH780	96.6% (0.82–1.00)	100% (0.99–1.00)	99.7% (0.98–1.00)	0.981(0.943–1.000)
BC-6000 Plus-O	100% (0.88–1.00)	99.7% (0.98–1.00)	99.7% (0.98–1.00)	0.981(0.945–1.000)
BC-6000 Plus-I	100% (0.88–1.00)	99.3% (0.98–1.00)	99.4% (0.98–1.00)	0.963(0.913–1.000)
Sysmex XN-9000-O	100% (0.88–1.00)	100% (0.99–1.00)	100% (0.98–1.00)	1.000(1.000–1.000)
Sysmex XN-9000-I	100% (0.88–1.00)	99.3% (0.98–1.00)	99.4% (0.98–1.00)	0.963(0.913–1.000)
Sysmex XN-9000-F	96.6% (0.82–1.00)	100% (0.99–1.00)	99.7% (0.98–1.00)	0.981(0.943–1.000)
Hand counting	89.7% (0.73–0.98)	99.3% (0.98–1.00)	98.5% (0.96–1.00)	0.904(0.821–0.987)

## 4. Conclusions

Platelet count plays an indispensable role in the treatment of many clinical diseases, such as atherosclerosis, thromboembolism, leukemia, and cancer [13–15]. Low platelet counts can affect the speed of vascular repair and cause bleeding [16], while high-value platelets can easily induce thrombus formation [17]. Accurate counting of platelets is very important for clinicians in selecting theraputic strategies and monitoring medications. However, there are many factors influencing platelet count, such as large platelets, small red blood cells, and platelet aggregation [18]. Moreover, Kim et al. found that inaccurate platelet counts can lead to misdiagnosis of disseminated intravascular coagulation (DIC) [15]. Hence, our study used IRM as the gold standard to compare the accuracy of several platelet detection methods (LH780, BC-6000 Plus-O, BC-6000 Plus-I, Sysmex XN-9000-O, Sysmex XN-9000-I, Sysmex XN-9000-F, and hand counting) which are commonly used in China.

The superiority between the optical and the impedance methods used in the automatic blood analyzers has been a frequently debated topic [19]. Furthermore, a new fluorescence method for platelet detection has aroused the attention of scholars [20]. In the current study, we evaluated the overall level of platelets and divided patients into three levels (thrombocytopenia, normal, and thrombocytosis) based on the clinical significance of platelet levels. Our study found that the correlation coefficient between Sysmex XN-9000-F (r = 0.988) and IRM was larger than that of Sysmex XN-9000-O (r = 0.984) or Sysmex XN-9000-I (r = 0.937) for thrombocytopenic samples. Compared with IRM, the average bias of Sysmex XN-9000-O and Sysmex XN-9000-F was the same, but the 95% LA of Sysmex XN-9000-O (95% LA = -10.8 to +7.8) was wider than Sysmex XN-9000-F (95% LA = -9.4 to +6.4) for thrombocytopenic samples. Schoorl et al. [20] found that the fluorescence method is more suitable for platelet count in patients with thrombocytopenia compared with optical and impedance methods, which was consistent with our findings.

Tantanate et al. [7] revealed that fluorescence was better than optical and impedance methods for counting platelets for samples with thrombocytosis. However, our results showed that the impedance method was superior to optical and fluorescence methods in the thrombocytosis group. The differences between our results and those of Tantanate et al. may be due to differences in study design. For instance, Tantanate et al. used samples from thalassemia patients, but we did not restrict to a specific disease population. Furthermore, Tantanate et al. [7] found that optical methods were more consistent with the reference method IRM than impedance methods for counting platelets at normal levels, which was also inconsistent with our results. Our results demonstrated that in normal platelet levels, the consistency of the impedance method was higher than the optical method, whether in a Sysmex XN-9000 instrument or a BC-6000 Plus instrument. The reason for the inconsistency may be due to the different people selected and the different types of instruments.

Due to the fact that different platelet detection principles are applied in these hematology analyzers, the flagging for platelet and red blood cell showed different outcomes. In LH780, impedance method is used to detect the size or volume of each platelet passing through the small hole, thereby obtaining the number of platelets. Since the impedance method only detects the volume, it is inaccurate in counting big platelets, small red blood cells, platelet aggregation, and cell debris. In BC-6000Plus and XN-9000, nucleic acid fluorescence staining method is used when impedance platelet is not accurate, the fluorescent dye stains RNA and part of DNA in cells, and finally distinguishes different substances by the intensity of light signals. This kind of method can avoid the influence of small red blood cells and red blood cell fragments on platelet counting. In the present study, a total of 77 samples were excluded, and the reasons for the instrument alarm were as follows: 13 cases of platelet aggregation specimens, 19 cases of cell fragments specimens, 3 cases of small red blood cell specimens and 16 cases of abnormal platelet histograms. In addition, 26 cases of interfering substances were found in peripheral blood cell smear. Therefore, in clinical laboratory work, careful peripheral blood cell smear examination should be performed in time and combined with the principle of each instrument channel to review the specimens to avoid reporting inaccurate platelet count results to clinicians.

In order to explore the precision of Beckman Coulter LH780, Sysmex XN-9000 and IRM, the CV values were calculated. The CV of LH780 in our study was 8.75%, which was very similar to the results of Harrison et al. (CV of LH750 = 8.78%) [21]. However, the CV value of IRM in our study was 1.51%, which was inconsistent with that of Harrison et al. (CV of IRM = 5.65%) [21]. This disagreement may be due to the differences in the number of samples and platelets in each sample. Few studies [22, 23] have evaluated the platelet counts by Mindray BC-6000 Plus or hand counting. Our results found that BC-6000 Plus-I had a CV value of 3.26% while hand counting showed a low precision with a CV value of 18.39%.

All evaluated methods had acceptable sensitivity, specificity, and coincidence rates for the diagnosis of thrombocytopenia and thrombocytosis, which was consistent with the findings of Tantanate et al. [7]. Thus, all assessed methods can be used for screening and exclusion of patients with thrombocytopenia and thrombocytosis. Moreover, in the present study, Sysmex XN-9000-O (Kappa value = 1.000) showed a high degree of agreement with IRM at high platelet levels, while Sysmex XN-9000-F (Kappa value = 0.994) performed commendably at low platelet levels. However, the fluorescence method was not suitable for routine counting of platelets due to its expense.

Barbara et al. [24] used 23 different types of automated blood analyzers to compare the accuracy of the thrombocytopenia count and found that most of the automated blood analyzers produced higher results than IRM. Our findings are consistent with those of Barbara et al [24]. Our study has the following advantages: our research use a variety of blood automatic analyzers (LH780, BC-6000 Plus-O, BC-6000 Plus-I, Sysmex XN-9000-O, Sysmex XN-9000-I and Sysmex XN-9000-F) and hand counting to analyze their performance on platelet counts. Our study analyzes the degree of agreement between each counting method (LH780, BC-6000 Plus-O, BC-6000 Plus-I, Sysmex XN-9000-O, Sysmex XN-9000-I, Sysmex XN-9000-F and hand counting) and the reference method using different levels of platelet count (thrombocytopenia, normal, and thrombocytosis).

The limitations of our study are summarized in the following points: First, only 29 patients with high platelet count were included. Second, we only evaluated the LH780, BC-6000 Plus, Sysmex XN-9000 and hand counting. Finally, our research was a single-center study. Therefore, our research requires a large-scale multi-center analysis of multiple types of blood automated analyzers to verify the reliability of our results.

In summary, Sysmex XN-9000-F has superior correlation and diagnosis efficacy for thrombocytopenic samples. BC-6000 Plus-I is the best method that has high association and consistency with IRM for samples in normal platelets range. For samples with thrombocytosis, Sysmex XN-9000-I has the highest correlation with IRM, while Sysmex XN-9000-O has the best diagnosis efficacy. Therefore, in the case of thrombocytosis, the combination of Sysmex XN-9000-I and Sysmex XN-9000-O should provide more accurate platelet count.

## Supporting information

S1 Fig Spearman rank correlation coefficient of evaluated methods from the IRM for thrombocytopenic samples. A. Spearman rank correlation coefficient: LH780 & IRM, B. Spearman rank correlation coefficient: BC-6000Plus-O & IRM, C. Spearman rank correlation coefficient: BC-6000Plus-I & IRM, D. Spearman rank correlation coefficient: XN-9000-O & IRM, E. Spearman rank correlation coefficient: XN-9000-I & IRM, F. Spearman rank correlation coefficient: XN-9000-F & IRM, G. Spearman rank correlation coefficient: Hand counting & IRM.
(JPG)

S2 Fig Spearman rank correlation coefficient of evaluated methods from the IRM for samples with platelet counts in the normal range. A. Spearman rank correlation coefficient: LH780 & IRM, B. Spearman rank correlation coefficient: BC-6000Plus-O & IRM, C. Spearman rank correlation coefficient: BC-6000Plus-I & IRM, D. Spearman rank correlation coefficient: XN-9000-O & IRM, E. Spearman rank correlation coefficient: XN-9000-I & IRM, F. Spearman rank correlation coefficient: XN-9000-F & IRM, G. Spearman rank correlation coefficient: Hand counting & IRM.
(JPG)

S3 Fig Spearman rank correlation coefficient of evaluated methods from the IRM for samples with thrombocytosis. A. Spearman rank correlation coefficient: LH780 & IRM, B. Spearman rank correlation coefficient: BC-6000Plus-O & IRM, C. Spearman rank correlation coefficient: BC-6000Plus-I & IRM, D. Spearman rank correlation coefficient: XN-9000-O & IRM, E. Spearman rank correlation coefficient: XN-9000-I & IRM, F. Spearman rank correlation coefficient: XN-9000-F & IRM, G. Spearman rank correlation coefficient: Hand counting & IRM.
(JPG)

S4 Fig Bland-Altman plots showing the average bias and 95% limits of agreement of evaluated methods from the IRM for thrombocytopenic samples. A. Bland-Altman difference plots: LH780 vs IRM, B. Bland-Altman difference plots: BC-6000Plus-O vs IRM, C. Bland-Altman difference plots: BC-6000Plus-I vs IRM, D. Bland-Altman difference plots: XN-9000-O vs IRM, E. Bland-Altman difference plots: XN-9000-I vs IRM, F. Bland-Altman difference plots: XN-9000-F vs IRM, G. Bland-Altman difference plots: Hand counting vs IRM.
(JPG)

S5 Fig Bland-Altman plots showing the average bias and 95% limits of agreement of evaluated methods from the IRM for samples with platelet counts in the normal range. A. Bland-Altman difference plots: LH780 vs IRM, B. Bland-Altman difference plots: BC-6000Plus-O vs IRM, C. Bland-Altman difference plots: BC-6000Plus-I vs IRM, D. Bland-Altman difference plots: XN-9000-O vs IRM, E. Bland-Altman difference plots: XN-9000-I vs IRM, F. Bland-Altman difference plots: XN-9000-F vs IRM, G. Bland-Altman difference plots: Hand counting vs IRM.
(JPG)

S6 Fig Bland-Altman plots showing the average bias and 95% limits of agreement of evaluated methods from the IRM for samples with thrombocytosis. A. Bland-Altman difference plots: LH780 vs IRM, B. Bland-Altman difference plots: BC-6000Plus-O vs IRM, C. Bland-Altman difference plots: BC-6000Plus-I vs IRM, D. Bland-Altman difference plots: XN-9000-O vs IRM, E. Bland-Altman difference plots: XN-9000-I vs IRM, F. Bland-Altman difference plots: XN-9000-F vs IRM, G. Bland-Altman difference plots: Hand counting vs IRM.
(JPG)

S1 File Dataset. Raw data.
(XLS)
