# Supplementary figures and images for: Compare the accuracy and precision of Coulter LH780, Mindray BC-6000 Plus, and Sysmex XN-9000 with the international reference flow cytometric method in platelet counting
Source: PLoS One. 2019 May 24;14(5):e0217298. doi: 10.1371/journal.pone.0217298 (PMC6534315; doi:10.1371/journal.pone.0217298)

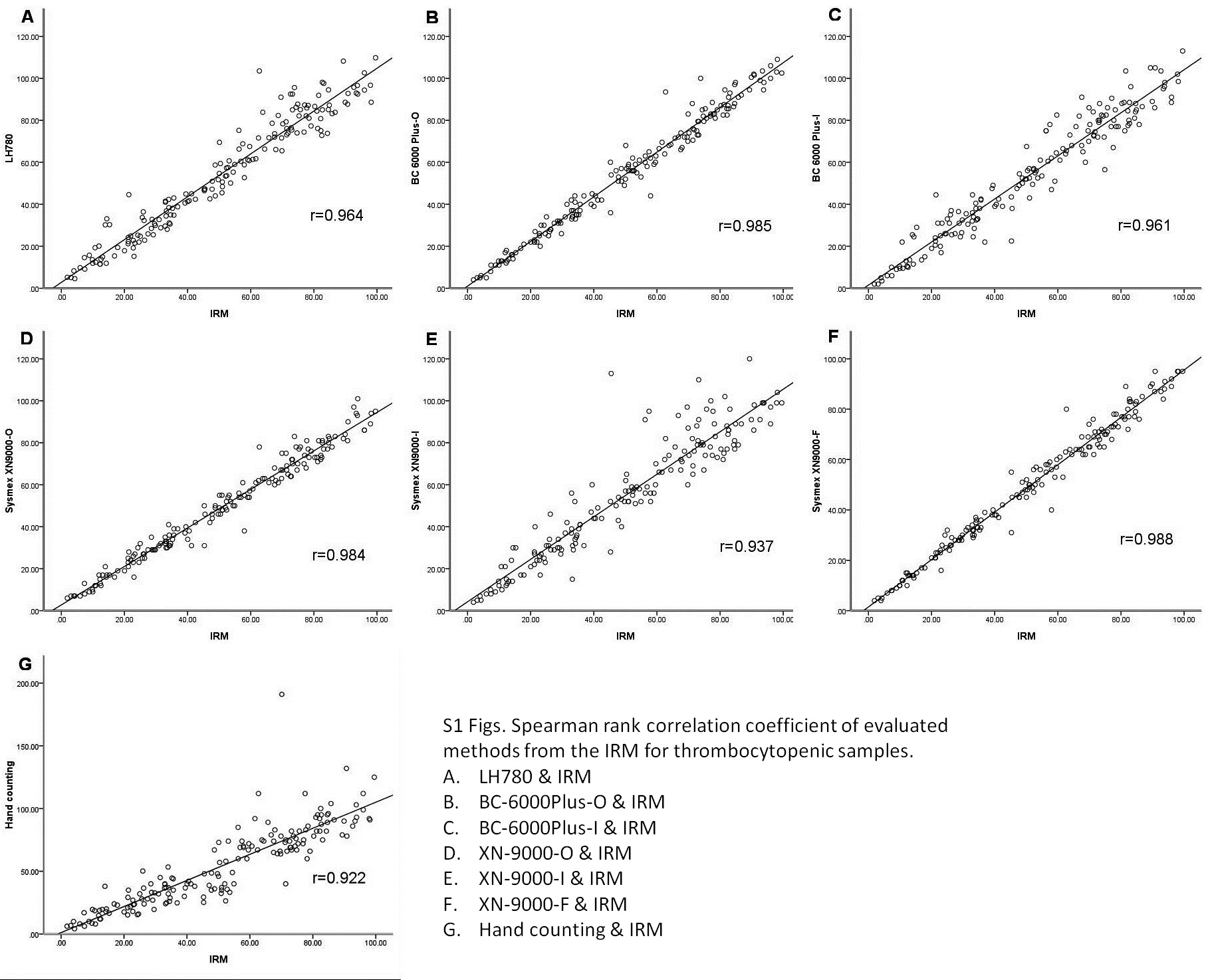

Supplement: S1 Fig — A. Spearman rank correlation coefficient: LH780 & IRM, B. Spearman rank correlation coefficient: BC-6000Plus-O & IRM, C. Spearman rank correlation coefficient: BC-6000Plus-I & IRM, D. Spearman rank correlation coefficient: XN-9000-O & IRM, E. Spearman rank correlation coefficient: XN-9000-I & IRM, F. Spearman rank correlation coefficient: XN-9000-F & IRM, G. Spearman rank correlation coefficient: Hand counting & IRM. (JPG) [file pone.0217298.s001.jpg]

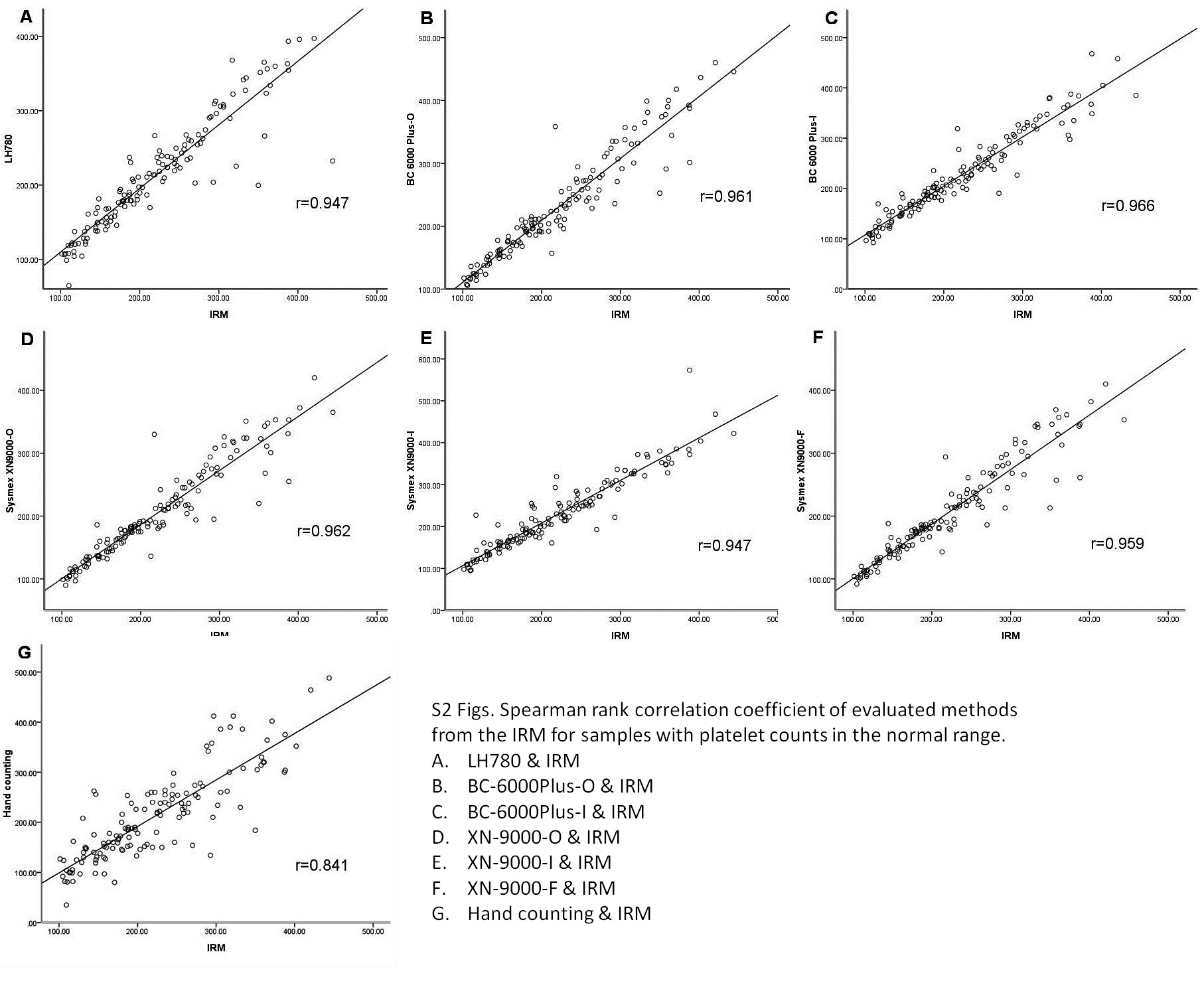

Supplement: S2 Fig — A. Spearman rank correlation coefficient: LH780 & IRM, B. Spearman rank correlation coefficient: BC-6000Plus-O & IRM, C. Spearman rank correlation coefficient: BC-6000Plus-I & IRM, D. Spearman rank correlation coefficient: XN-9000-O & IRM, E. Spearman rank correlation coefficient: XN-9000-I & IRM, F. Spearman rank correlation coefficient: XN-9000-F & IRM, G. Spearman rank correlation coefficient: Hand counting & IRM. (JPG) [file pone.0217298.s002.jpg]

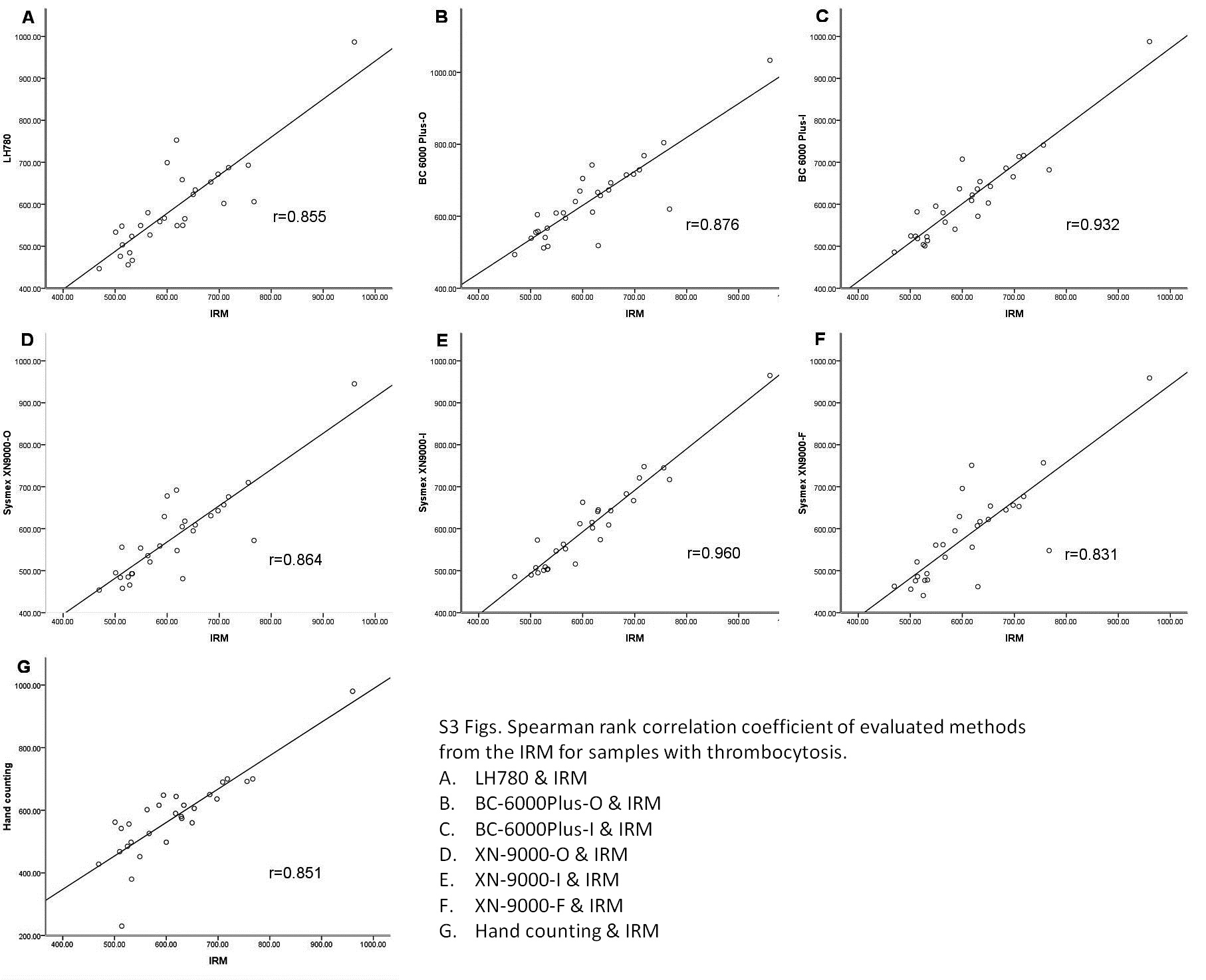

Supplement: S3 Fig — A. Spearman rank correlation coefficient: LH780 & IRM, B. Spearman rank correlation coefficient: BC-6000Plus-O & IRM, C. Spearman rank correlation coefficient: BC-6000Plus-I & IRM, D. Spearman rank correlation coefficient: XN-9000-O & IRM, E. Spearman rank correlation coefficient: XN-9000-I & IRM, F. Spearman rank correlation coefficient: XN-9000-F & IRM, G. Spearman rank correlation coefficient: Hand counting & IRM. (JPG) [file pone.0217298.s003.jpg]

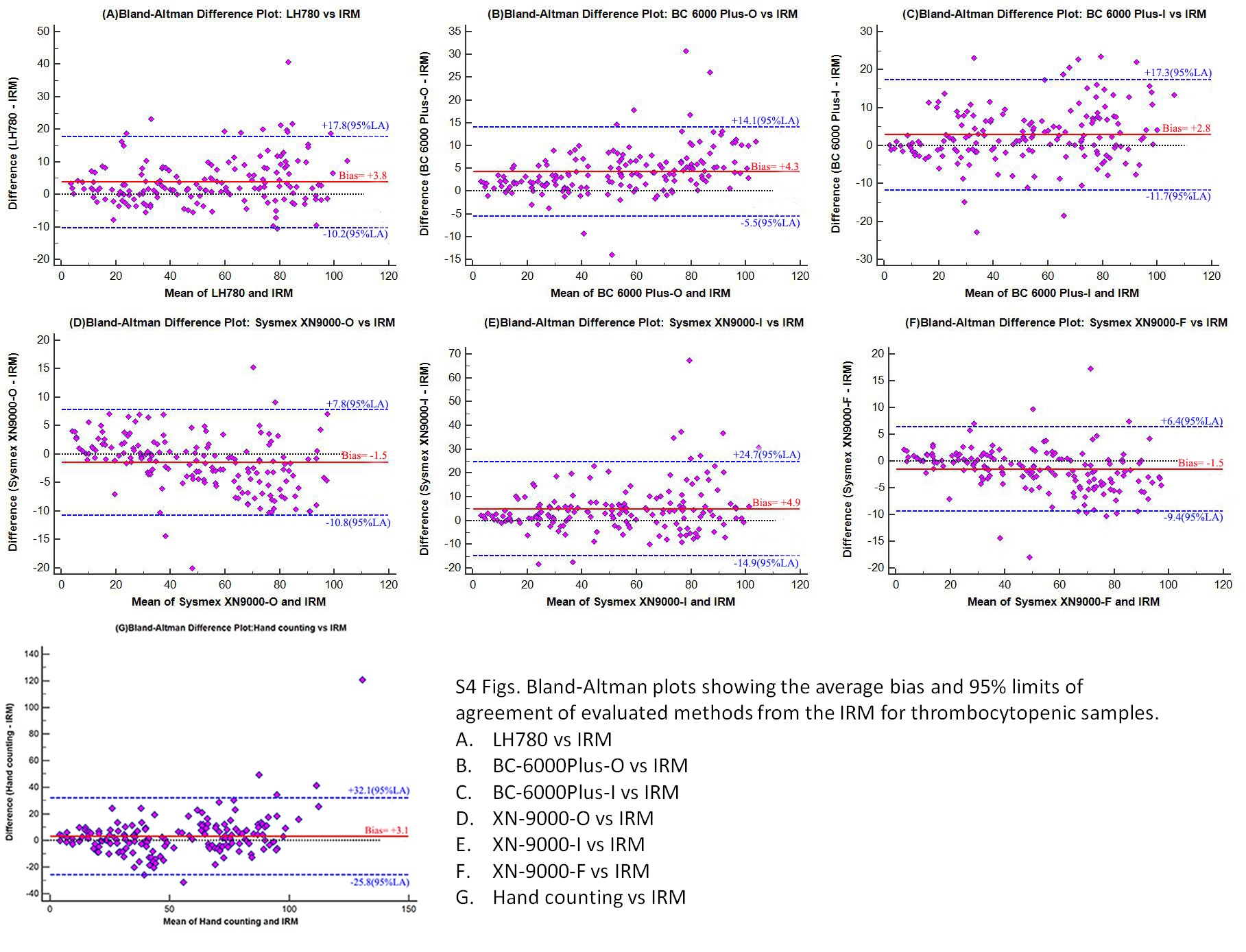

Supplement: S4 Fig — A. Bland-Altman difference plots: LH780 vs IRM, B. Bland-Altman difference plots: BC-6000Plus-O vs IRM, C. Bland-Altman difference plots: BC-6000Plus-I vs IRM, D. Bland-Altman difference plots: XN-9000-O vs IRM, E. Bland-Altman difference plots: XN-9000-I vs IRM, F. Bland-Altman difference plots: XN-9000-F vs IRM, G. Bland-Altman difference plots: Hand counting vs IRM. (JPG) [file pone.0217298.s004.jpg]

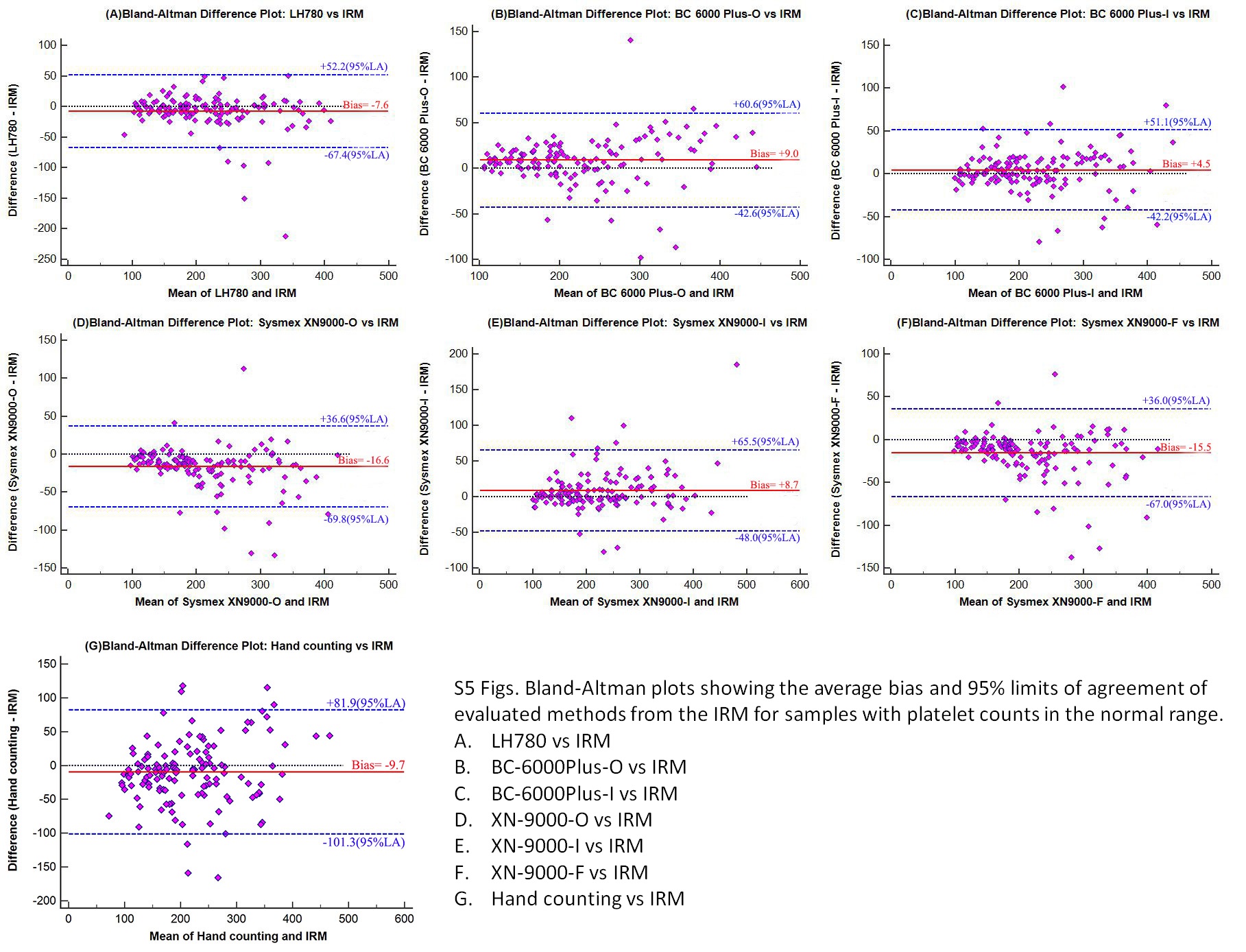

Supplement: S5 Fig — A. Bland-Altman difference plots: LH780 vs IRM, B. Bland-Altman difference plots: BC-6000Plus-O vs IRM, C. Bland-Altman difference plots: BC-6000Plus-I vs IRM, D. Bland-Altman difference plots: XN-9000-O vs IRM, E. Bland-Altman difference plots: XN-9000-I vs IRM, F. Bland-Altman difference plots: XN-9000-F vs IRM, G. Bland-Altman difference plots: Hand counting vs IRM. (JPG) [file pone.0217298.s005.jpg]

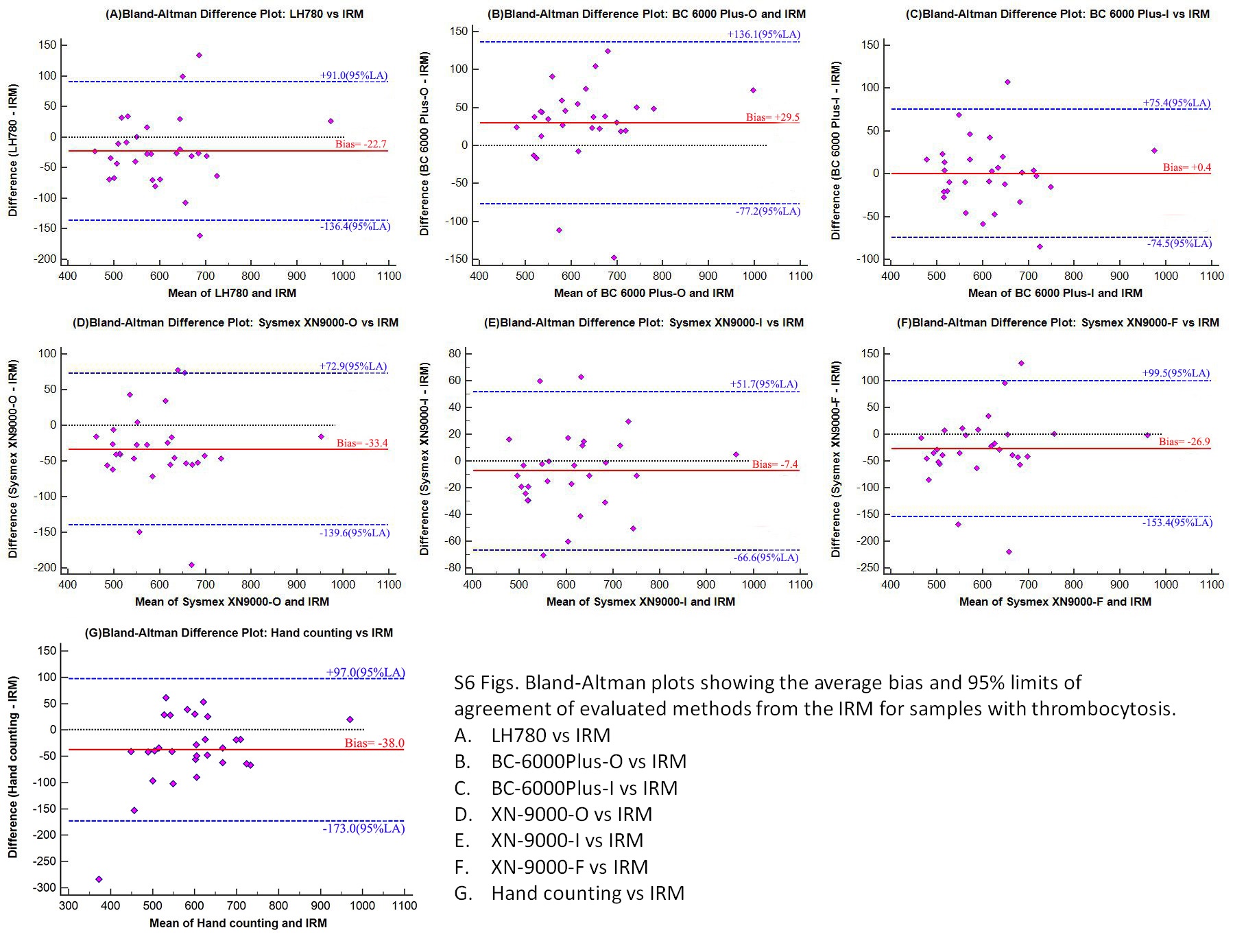

Supplement: S6 Fig — A. Bland-Altman difference plots: LH780 vs IRM, B. Bland-Altman difference plots: BC-6000Plus-O vs IRM, C. Bland-Altman difference plots: BC-6000Plus-I vs IRM, D. Bland-Altman difference plots: XN-9000-O vs IRM, E. Bland-Altman difference plots: XN-9000-I vs IRM, F. Bland-Altman difference plots: XN-9000-F vs IRM, G. Bland-Altman difference plots: Hand counting vs IRM. (JPG) [file pone.0217298.s006.jpg]
